# Evaluation of the radiosensitizing effect of MEK inhibitor KZ-001 on non-small cell lung cancer cells in vitro

**DOI:** 10.2478/abm-2023-0064

**Published:** 2023-10-26

**Authors:** Gongchao Huang, Wenqin Zhang, Hongqi Tian

**Affiliations:** 1 Department of Chemistry, School of Science, Tianjin University, Tianjin 300072, China; 2 Shanghai Kechow Pharma, Inc., Shanghai 201203, China

**Keywords:** apoptosis, DNA damage, MEK inhibitor, non-small cell lung cancer, radiosensitization

## Abstract

**Background:**

Non-small cell lung cancer (NSCLC) has a poor prognosis and usually presents resistance against radiotherapy. MEK inhibitors have been proven to possess a radiosensitization effect. The compound KZ-001 as a particular MEK inhibitor is superior to the listed MEK inhibitor AZD6244.

**Objective:**

To investigate whether KZ-001 could enhance the radiosensitivity of NSCLC cell lines in vitro.

**Methods:**

MTT and colony formation assay were used to evaluate the radiosensitivity effect of KZ-001. Immunofluorescence, cell cycle, apoptosis staining, and western blot experiments were used to explore the radiosensitivity mechanism.

**Results:**

KZ-001 significantly decreased A549 cell viability at 6 Gy and 8 Gy radiation doses and caused the radiosensitivity at 1 Gy, 4 Gy, and 6 Gy in colony formation experiments. The A549 apoptosis ratio induced by irradiation (IR) combined with KZ-001 increased significantly in comparison with that by IR monotherapy (10.57% vs. 6.23%, *P* = 0.0055). The anti-apoptosis marker Bcl-XL was found downregulated in KZ-001 and IR-treated A549/H460 cells, but apoptosis marker Bax was downregulated in H460. Extracellular regulated protein kinases (ERK1/2) phosphorylation of H460 cells could be blocked both by IR alone and IR combined with KZ-001. IR combined with KZ-001 is able to inhibit ERK activation of A549 cells apparently. KZ-001 increased the proportion of G2 phase in irradiated cells from 21.24% to 32.22%. KZ-001 could also significantly increase the double-strand break damage cell ratio to more than 30% compared to the irradiation alone group.

**Conclusions:**

MEK1/2 inhibitor KZ-001 is a potential radiosensitizer for clinical applications.

Researchers report cancer as the worldwide leading cause of death. An estimated 609,360 people in the US will die from cancer in 2022, corresponding to almost 1,700 deaths/d. The greatest number of deaths are from cancers of the lung, prostate, and colorectum in men and of the lung, breast, and colorectum in women. More than 350 people will die each day from lung cancer, which is more than the corresponding numbers for breast, prostate, and pancreatic cancers combined [1]. Population aging and changing lifestyle are the expected causes for rapidly increasing cancer cases and deaths [2]. Conventional treatments for cancer include surgery, chemotherapy, radiotherapy, immunotherapy, and targeted therapy [3]. In terms of control via conventional cancer treatments, radiotherapy can achieve histologically local control in various cancer types by producing ROS and inducing DNA damage. Despite using advanced radiotherapy, the radiation from radiotherapy often affects the normal tissues [4, 5]. Simultaneously, resistance to radiation often develops in patients receiving radiotherapy, which is one of the main obstacles to cancer treatment.

According to studies, inhibition of *RAS* activation can lead to radiosensitivity in *RAS*-mutated rodents and human tumor cell lines [6]. Thirty percent of cancer cases possess active mutations in the *RAS* gene; however, *KRAS* is the most common and usually obtained in the early stages of tumorigenesis. Moreover, studies have confirmed that the extracellular regulated protein kinases (ERK) activation in endothelial cells increases the survival rate of irradiated cells.

The mitogen-activated protein kinase (MAPK) signaling pathway activity changes the expression of proteins that regulate the adhesion, movement, differentiation, and proliferation of tumor cells; the pathway becomes a significant molecular target for ionizing radiation therapy [7, 8].

MEK is the central downstream component of *RAS* and essential for ERK phosphorylation [9]. MAPK pathway blocked by MEK1/2 inhibitors can clinically benefit cancer treatments. It has been reported that researchers have performed radiosensitization studies with MEK inhibitors. Chung et al. [10] explored and evaluated the radiosensitization using AZD6244, a second-generation MEK inhibitor. AZD6244 was found to increase the radiosensitivity of tumor cells (A549, MiaPaCa2, and DU145), with a dose enhancement factor (DEF) at a 0.10 surviving fraction of 1.16–2.0. In 2014, Xia et al. [11] reported the radiosensitization effect and mechanism of MEK-specific inhibitor U0126 in A549 cells. The study of Estrada-Bernal et al. [12] shows the mechanism of MEK inhibitor GSK1120212 in radiosensitization of pancreatic cancer cells. Also, Zhu and Tian [13] found the radiosensitization effect and mechanism of novel benzothiadiazole derivatives in *KRAS*-mutated (*KRAS*m) non-small cell lung cancer (NSCLC).

KZ-001 is a small-molecular MEK inhibitor developed by us, which is still in the early stage [14]. Preclinical studies have shown that the efficacy of KZ-001 is 30-fold greater than that of AZD6244 in *BRAF-* and *KRAS*-mutated tumor cell lines. This study mainly aims to explore and evaluate the radiosensitization effect and mechanism of KZ-001, a MEK inhibitor, in *RAS*-mutated tumor cells. We expect the study results to theoretically support the clinical treatment using MEK inhibitors combined with radiotherapy and provide a treatment strategy for cancer patients.

## Material and methods

### Cell culture and proliferation assay

**Figure 1.**
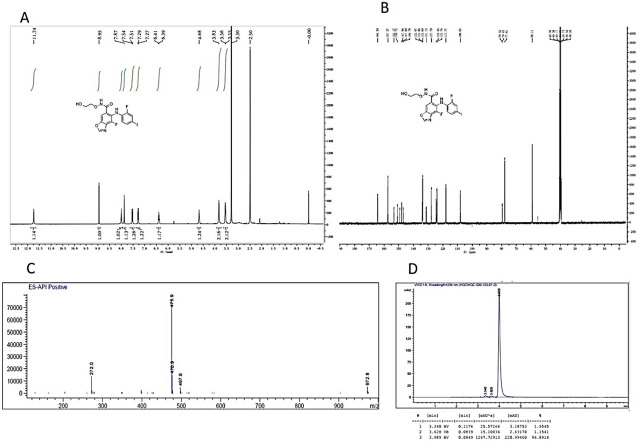
Characterization and purity of the KZ-001. **(A)** ^1^H NMR spectra of KZ-001; **(B)** ^13^C NMR spectra of KZ-001; **(C)** MS spectra of KZ-001; and **(D)** HPLC spectra of KZ-001.

A549 NSCLC cells (Chinese Academy of Medical Sciences and Peking Union Medical College) were cultured in F12 (Gibco, C11765500BT) medium supplemented with 10% fetal bovine serum (FBS) (Yeasen, 40130ES76), and H460/H441 was maintained in a 1640 (Gibco) medium supplemented with 10% FBS (Yeasen, 40130ES76). Both culture media contained 1% penicillin and streptomycin (Solarbio, P1400). Cellular proliferation assay analysis was conducted using 3-(4,5-dimethylthiazole-2-yl)-2,5-diphenyl-2H-tetrazolium bromide (MTT) (Solarbio, M1080). In brief, the cells were seeded in 96-well plates (Corning, 3599) at an appropriate density in a 90 μL cell culture medium until attached. Then, KZ-001 (Kechow; the characterization and purity of the KZ-001 are indicated in **Figure 1**) at 1 mM was gradient-diluted with DMSO at a 1:10 ratio. Finally, the assay was prepared with a 10-fold concentration in 5% DMSO media. The cells were treated either with KZ-001 alone, irradiation at different doses, or KZ-001 combined with irradiation. The irradiation was carried out using a Pantak (Solon) X-ray source at a dose rate of 1.55 Gy/min. After treatment, MTT (0.5 mg/mL) was added to the culture plate and incubated for 4 h. Then, the culture medium was discarded and 150 μL DMSO was added. The absorbance was measured at 570 nm using a microplate reader (Utrao, SM600).

### Western blot

RIPA buffer (R0020), protein phosphatase inhibitor (all-in-one, 100×) (P1260), and BCA protein assay kit (PC0020) were purchased from Solarbio. In addition, NuPage 10% Bis-Tris Gel (NP0301BOX) was purchased from Invitrogen. PVDF membrane (Immobilon-P) and eECL western blot kit (CW0049) were from Millipore and CWBIO, respectively. Further, anti-p-ERK1/2 antibody (4370) and anti-ERK1/2 (4695) antibody were purchased from Cell Signaling Technology. Anti-Bcl-xl antibody (ab32370) and anti-Bax antibody (ab32503) were purchased from Abcam. Finally, anti-GAPDH antibody (HRP-60004) was purchased from ProteinTech.

The cells were seeded at an appropriate density (80%–90% confluency). After being subject to treatment with 6 Gy irradiation, KZ-001 at 100 nM, or 6 Gy in combination with KZ-001 at 100 nM, respectively, the cell lysates were extracted and collected using radioimmunoprecipitation assay (RIPA) buffer containing protein phosphatase inhibitor (all-in-one, 100×). As a control, the cells were treated with a 0.1% DMSO medium.

Equal quantities of total protein from cell were resolved on NuPage 10% Bis-Tris Gel and then transferred to the PVDF membrane. The proteins of interest were blotted with the indicated antibodies (anti-p-ERK1/2 ab; anti-ERK1/2 ab; anti-Bcl-xl ab; and anti-Bax ab). As a final step, the chemiluminescence was generated with an eECL western blot kit and detected with the use of a Bio-Rad ChemiDoc XRS+ System.

### Clonogenic survival assay

The A549 and H460 (Chinese Academy of Medical Sciences and Peking Union Medical College) cells were trypsinized to generate a single-cell suspension and were then seeded in 6-well plates for 14 h to allow attachment before treatment. Each treatment condition had 3 replicates. After treating by vehicle (dimethylsulfoxide) or KZ-001 (100 nM) for 1–2 h, the cells were treated with 0 Gy, 1 Gy, 2 Gy, 4 Gy, or 6 Gy of radiation. The drugs were washed out after 24 h; next, the cells were maintained for 2 weeks. The colonies thus obtained were fixed and stained with the use of 0.1% crystal violet (Solarbio, C8470) in 20% methanol. All the colonies consisting of at least 50 cells were counted. Finally, the data were analyzed using GraphPad Prism 8.0 (GraphPad Software, Inc.).

### Apoptosis assay

A549 and H460 cells at a density of 2 × 10^5^/well were inoculated into the wells of 6-well plates and cultured overnight. Next, 100 nM KZ-001 or vehicle was added to the indicated wells. Each treatment was triplicated. Subsequently, 100 nM KZ-001 was added to each well, and the vehicle control group was treated with a 0.5% DMSO culture medium. Then, cells were exposed to IR at a dose of 6 Gy. After 24 h, 48 h, and 72 h of treatment, the cells were digested and stained with fluorescein isothiocyanate (FITC), annexin V, and propediene iodide (PI) as per the instructions of apoptosis detection kit (YEASEN, 40301ES50) and analyzed with the use of flow cytometry (BD Biosciences, FACSCelesta).

### Cell cycle assay

A549 cells at a density of 1 × 10^5^ cells/mL were seeded into wells of the 6-well plates, cultured overnight, and then pretreated with 100 nM KZ-001 for 1 h. The vehicle control group was treated with a 0.5% DMSO culture medium. Then, these pretreated cells were exposed to IR at a 6 Gy dose. After 48 h incubation, the cells were digested and washed with PBS, fixed in 70% ice-cold ethanol at 4 °C overnight, and then processed in compliance with the instructions accompanying the cell cycle analysis kit (Yeasen, 40301). Ultimately, the fluorescence intensity of each sample was measured using flow cytometry (BD Bioscience, FACSCelesta) and analyzed with ModFit LT software.

### Immunofluorescence staining

A549 cells at a density of 5 × 10^5^/well were seeded into wells of 12-well plates with coverslips (Solarbio, YA0351) and cultured overnight. The next day, the cells were pretreated with 100 nM KZ-001 or 0.5% DMSO for 1 h, and then irradiated with a 6 Gy dose. After 48 h, the cells were fixed with a 10% neutral buffered formalin (Solarbio, G2161) at 37 °C for 30 min and permeabilized with Triton X-100 (Solarbio, P1080) for 30 min at room temperature. After being blocked with PBS containing 1% bovine serum albumin (Solarbio, A8010) at room temperature for 60 min, the samples were incubated with primary rabbit anti-gamma H2AX (phospho S139) antibody (Abcam, ab81299; 1:250) at 4 °C overnight followed by the anti-rabbit-PE secondary antibody (Proteintech, SA00008-2; 1:50) at room temperature for 1.5 h in the dark.

### Statistical analyses

Statistical analyses were performed with the use of GraphPad Prism 8.0 (GraphPad Software, Inc.), using *t* test analysis between 2 independent groups. *P* values were calculated to visually indicate the statistical results, and the significance was defined as follows: *P < 0.05, **P < 0.01, and ***P < 0.001.

## Results

### Optimization of KZ-001 concentration and irradiation dose

**Figure 2.**
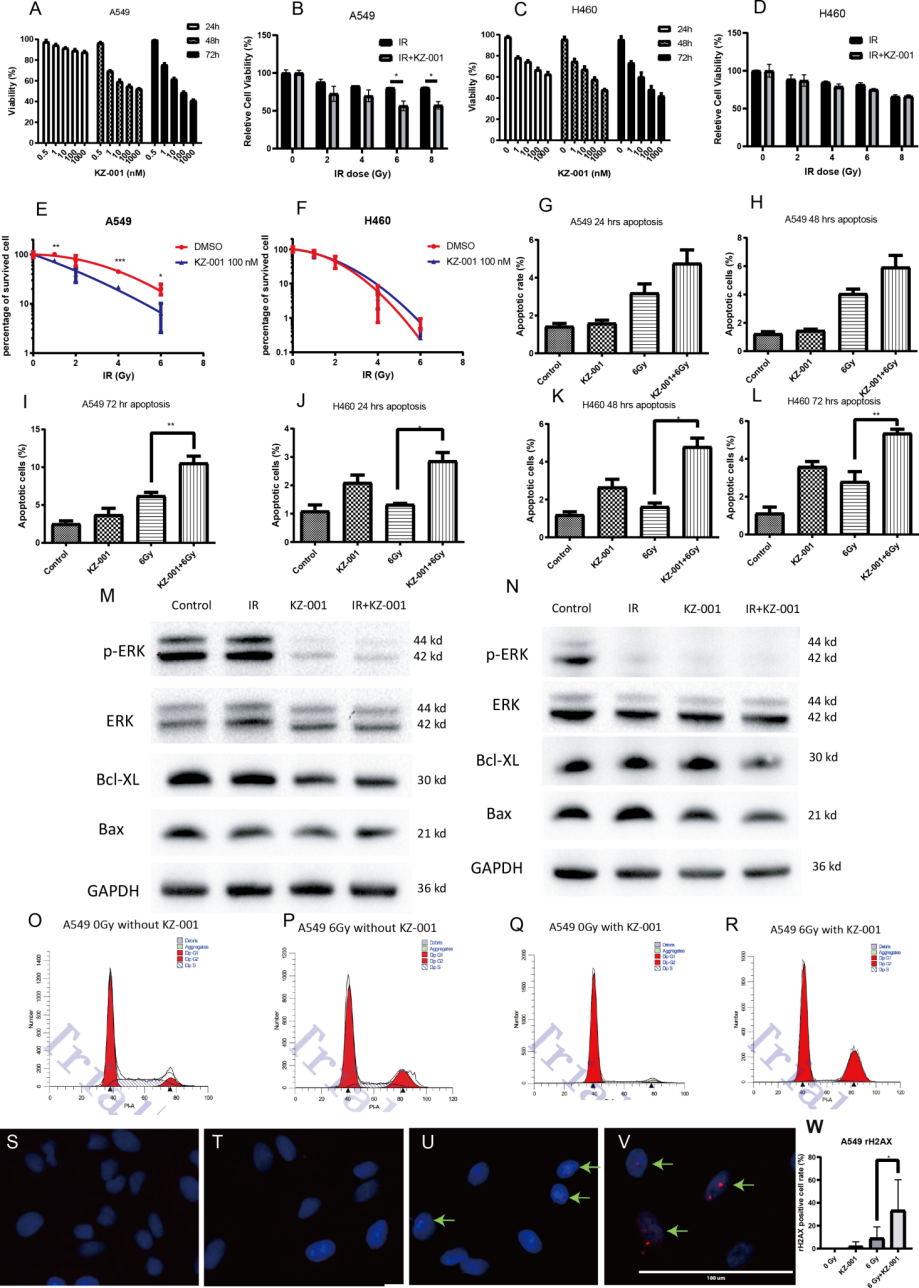
Cell viability under the treatment of IR and KZ-001 **(A–D)**. Concentration screening of KZ-001 in A549 cells **(A)** and in H460 cells **(C)**; irradiated A549 **(B)** and H460 **(D)** cell viability comparison between vehicle and KZ-001 treated cells. KZ-001 + IR vs. IR group, *P < 0.05. **(E, F)** Evaluation of the KZ-001 effect on the proliferation of irradiated A549 **(E)** and H460 cells **(F)** by colony formation assay. A549 and H460 cells were seeded and then treated as indicated. For A549 cells, KZ-001 made the percentage of survival significantly decrease under radiation at the dose of 1 Gy, 4 GY, and 6 Gy, whereas the KZ-001 treated H460 cells did not show a decrease in viability compared with the vehicle control group. KZ-001 + IR vs. IR group, *P < 0.05, **P < 0.01, ***P < 0.001. **(G–L)** Effects of KZ-001 on the apoptosis level of irradiated A549 **(G–I)** and H460 **(J–L)** cells at different times. A549 and H460 cells were inoculated into 6-well plates at a proper density, and cells were cultured overnight, followed by exposure to 6 Gy IR with or without KZ-001 at 100 nM. After KZ-001 treatment for 24 h, 48 h, and 72 h, cells were harvested and tested with the use of an apoptosis detection kit as the kit instruction indicated. KZ-001 + IR vs. IR group, *P < 0.05, **P < 0.01. **(M, N)** Effect of KZ-001 on the expression of pERK and apoptosis markers in irradiated A549 **(M)** and H460 **(N)** cells. Cells were seeded into 6-well plates at a proper density, then cells were treated with 6 Gy, KZ-001 100 nM, or 6 Gy combined with KZ-001 100 nM. After 24 h, cell lysate was extracted and analyzed. **(O–R)** Effect of KZ-001 on the cell cycle of an irradiated A549 cell line. A549 cells were seeded into wells of the 6-well plates, cultured overnight, and then pretreated with KZ-001. Then, cells were exposed to IR at a 6 Gy dose. After 48 h incubation, the cells were digested and processed in compliance with the cell cycle analysis kit (Yeasen, 40301) instructions. **(S–W)** Effect of KZ-001 on the level of DNA double-strand damage markers in irradiated A549 cells. A549 cells were seeded into wells of a 12-well plate with coverslips and cultured overnight. Then, cells were pretreated with 100 nM KZ-001 or 0.5% DMSO for 1 h, and then irradiated with a 6 Gy dose. After incubation for 48 h, each group of cells was fixed and stained with DSB-related antibodies. **(S)** Untreated A549 cells; **(T)** A549 cells was treated with KZ-001 at 100 nM for 48 h; **(U)** A549 cells was exposed to radiation at 6 Gy; the small red dots to which the green arrows point indicate that DNA DSB took place and that γH2AX was detected with antibody; **(V)** A549 cells was exposed to radiation at 6 Gy, and then treated with KZ-001 at 100 nM for 48 h. The small red dots became larger than that of the radiation group. **(W)** The bar chart shows the percentage of γH2AX-carried cells in each group, and the positive rate of the KZ-001 irradiation group was significantly higher than that of the irradiation group alone. DSB, double-strand break; ERK, extracellular regulated protein kinases.

We used different concentrations (0.5–1,000 nM) of KZ-001 to treat 3 different NSCLC cells for 24 h, 48 h, and 72 h. The results illustrated that KZ-001 had a strong inhibitory effect on the A549 and H460 proliferation for 72 h (**Figures 2A and 2C**). As observed, 100 nM KZ-001 inhibited 50% cell viability A549 and H460 after 72 h incubation. Hence, 100 nM was selected as the incubation concentration of KZ-001 for the subsequent irradiation experiments.

Next, we used the MTT method for a preliminary exploration of the radiosensitivity of the cells. **Figures 2B and 2D** illustrate that compared to the irradiation monotherapy group, the KZ-001 significantly decreased A549 cell viability at 6 Gy and 8 Gy radiation doses; however, KZ-001 could not reduce the viability of irradiated H460 cells. Hence, for subsequent experiments, we chose 6 Gy as the irradiation dose for NSCLC.

### Effect of KZ-001 on the irradiated cell clone formation

Two NSCLC cell lines, A549 and H460, were pretreated with 100 nM KZ-001 for 1 h before irradiation. After 2 weeks, the number of clones was counted. Results showed that KZ-001 caused the radiosensitivity under 1 Gy, 4 Gy, and 6 Gy irradiation doses. However, KZ-001 did not aggravate the radiation impairment of H460 under any irradiation doses (**Figures 2E and 2F**). The results are consistent with the results of MTT analysis.

### Evaluation of the KZ-001 effect on irradiated cell apoptosis

We used annexin V and PI double staining method after exposure to 6 Gy irradiation dose with or without KZ-001 treatment to detect A549 and H460 apoptosis. **Figures 2G–I** illustrate that compared to 24 h and 48 h, the A549 apoptosis rate reached a relatively high level after 72 h incubation. Interestingly, the A549 apoptosis ratio induced by IR combined with KZ-001 increased significantly in comparison with that by IR monotherapy (10.57% vs. 6.23%, *P* = 0.0055). However, a time-dependent manner was not observed in the H460 apoptosis (**Figures 2J–L**). The apoptotic rate under the IR and KZ-001 treatment for 72 h was 5.37%, which was significantly higher than the 2.80% apoptotic rate under the IR treatment group. This data may have statistical significance, but it lacks biological significance compared to the A549 apoptotic rate. In summary, we consider that KZ-001 can increase the radiation-mediated apoptotic rate of the 2 NSCLC cell lines, and A549 had a significant apoptotic response to KZ-001.

To further explore the mechanism of apoptosis at the molecular level, we detected the anti-apoptotic marker Bcl-XL and the apoptotic marker Bax in the A549 cells (**Figure 2M**) cells and found that after a single IR, the expression of Bcl-XL was not significantly decreased, but KZ-001 could weaken its expression. Furthermore, the expression of Bcl-XL in the irradiation group plus KZ-001 also decreased compared to that in the single irradiation group. This result indicates that KZ-001 can reverse the tolerance of A549 to IR and progress to apoptosis 24 h after irradiation. At the same time, the lack of significant changes in Bax suggests that the time span of 24 h may be too short to observe significant apoptosis. KZ-001 could significantly downregulate the expression of the anti-apoptotic protein Bcl-XL in H460 cells after irradiation (**Figure 2N**), that is, it could promote cell apoptosis. This was consistent with the significantly higher rate of apoptosis of H460 cells after 24 h compared to the IR group. On the other hand, through the analysis of the apoptosis marker Bax, we found that the expression of Bax in the IR combined with KZ-001 group decreased, suggesting that the progress of apoptosis was blocked, which could also explain why the H460 apoptosis rate increased only about 1.5% compared to the IR group.

### Effect of KZ-001 on ERK1/2 level of irradiated cells

Generally, one common property of *KRAS*-mutant NSCLC is the existence of the predominant *KRAS*-GTP in cytosol, which persistently activates downstream kinases, with the RAF/MEK/MAPK being the most important. ERK is a significant protein to regulate cell proliferation and survival. KZ-001 as a highly potent and selective MEK inhibitor can inhibit the ERK1/2 phosphorylation to a lower level; however, previous experiments found that KZ-001 could not inhibit the viability of irradiated H460 cells to a lower level, which raised our doubt about the KZ-001 inhibitory effect. Therefore, we tested the ERK1/2 expression under the dual treatment of IR and KZ-001. The results showed that both IR alone and IR combined with KZ-001 can block the H460 ERK1/2 phosphorylation, whereas IR cannot inhibit A549 ERK1/2 phosphorylation; besides, IR combined with KZ-001 can significantly inhibit ERK activation (**Figures 2M and 2N**). Therefore, KZ-001 can cause radiosensitization in the A549 cell line but not in the H460 cell line. This may be the reason why A549 cells treated with KZ-001 were more sensitive to irradiation.

### Evaluation of KZ-001 effect on the irradiated cell cycle

Since it was observed that the combination of KZ-001 with IR promoted the radiosensitivity on A549, we further investigated whether KZ-001 can affect the cell cycle arrest. In the experiment, A549 was pretreated with 100 nM KZ-001 for 1 h and then exposed to 6 Gy irradiation dose. After 48 h, the cells were collected, stained with PI, and examined for changes in the cell cycle distribution using a flow cytometer. The outcome showed that compared to control, the irradiation alone increased the proportion of cells in the G2 phase from 9.14% to 21.24%, and the combination of IR and KZ-001 could further increase the proportion to 32.22% (**Figures 2O–R**). The IR plus KZ-001 treatment caused A549 arrest in the G2 phase, the main period of homologous recombination repair after DNA damage. Therefore, we speculated that KZ-001 aggravated the IR-mediated DNA damage.

### Effect of KZ-001 on DNA double-strands in irradiated cells

IR could cause DNA damages to the tumor cells, and DNA double-strand breaks (DSBs) are the most crucial form of this damage. DSBs subsequently trigger the DNA repair mechanism. Activated γH2AX is a significant biomarker for the DNA repair process; hence, γH2AX (the number of phosphorylated γH2AX nuclear aggregates) activation extent can measure the degree of DNA damage caused by IR. Detection results of the phosphorylated γH2AX in cells showed that compared to the irradiation group, the KZ-001 could significantly increase the positive cell ratio to 33.4% (*P* < 0.05) (**Figures 2S–W**), suggesting the KZ-001 can aggravate the degree of DNA damage, thereby achieving the effect of increased radiosensitivity.

## Discussion

Lung cancer is the leading cause of cancer-related death worldwide, and NSCLC accounts for 85% of all lung cancers. NSCLC has a poor prognosis. In addition, NSCLC is resistant to radiotherapy, partly due to various genetic changes in NSCLC, including mutation, amplification, deletion, and fusion, which aggravates the signal pathway and physiological activity abnormalities [15, 16]. It is worth noting that 20%–30% of NSCLC has a *KRAS* gene mutation, and data show that the *KRAS* gene is a predictor of radiotherapy resistance [17, 18]. Although efforts are underway to target mutant *KRAS*, no drug can directly target the same. Therefore, the current treatment strategies mainly focused on the *KRAS* downstream effector molecule inhibition [19].

*RAS*-driven tumors are at least partly resistant to monotherapy with MEK inhibitors, due to the loss of negative regulatory feedback mediated by ERK1/2 [20]. Therefore, it is necessary to develop new and more effective treatments. Fortunately, studies have reported that the combination of MEK inhibitor and radiotherapy could significantly inhibit the survival rate of *KRAS*-mutant lung cancer cells, i.e., MEK inhibition could enhance radiosensitivity to cancer cells. The compound KZ-001 involved in this study is a selective MEK inhibitor, superior to the listed MEK inhibitors; therefore, we investigated whether KZ-001 could enhance the radiosensitivity of *KRAS*m lung cancer cell lines in vitro.

We evaluated the radiosensitivity effect of KZ-001 in 2 *KRAS*-mutant lung cancer cell lines A549 (*KRAS* G12S) and H460 (*KRAS* Q61H). Unexpectedly, results show that KZ-001 combined with radiotherapy could significantly reduce the survival rate of A549 cells, whereas the effect on the H460 cell survival rate was insignificantly different from that of radiation alone. Meanwhile, the clone formation experiment results also verified this result. In addition, the western blot experiment confirmed that KZ-001 combined with radiotherapy significantly inhibited the ERK phosphorylation and blocked the MAPK abnormal activation.

The findings of our study demonstrate that the 2 types of NSCLCs showed different radiosensitivity, and we next investigated the association between KZ-001 and IR-mediated apoptotic cell death in these NSCLCs. Interestingly, the KZ-001 with IR-induced apoptotic ratio in A549 and H460 was significantly increased in comparison with that induced by IR monotherapy alone. The H460 cell line apoptotic ratio is statistically significant, while it lacks biological significance compared to the A549 cell line apoptotic ratio. KZ-001-mediated downregulation of Bcl-XL could also support this phenomenon in both A549 and H460. Although we did not observe the upregulation of Bax within 24 h after the KZ-001 treatment in A549, the upregulation of Bax was inevitable with the prolongation of KZ-001 action.

DNA damage via ionizing radiation is one of the key approaches to kill cancer cells; in fact, DNA DSB is the most crucial type of DNA damage and the decisive factor for cell radiosensitivity. Histone γH2AX phosphorylation is a DNA damage marker, especially when DNA DSBs are involved [21]. We used fluorescence immunoassay to evaluate the KZ-001 effect on the level of DNA damage marker γH2AX. As expected, in A549 cell lines, the combination of KZ-001 and radiotherapy significantly increased the level of intracellular γH2AX phosphorylation compared to radiation alone.

DNA damage response can cause cell cycle arrest by activating cell cycle checkpoints, promoting DNA repair, and changing transcription, and ultimately maintaining cell survival. Studies have shown that CHK1 is phosphorylated and activated when DNA undergoes damage and blocks the cell cycle in the G2/M phase. Our results show that compared to the radiotherapy alone, the KZ-001 combined with radiotherapy had induced more cell cycle arrest in the G2/M phase, indicating that KZ-001 combined with radiotherapy had induced more DNA damage. This was consistent with the immunofluorescence results.

## Conclusions

This study shows that radiotherapy combined with MEK inhibitor KZ-001 can significantly block the MAPK pathway, reduce the ERK phosphorylation level, significantly increase DNA DSBs, increase cell cycle G2/M phase arrest, induce A549 apoptosis, and finally play a role of radiosensitizer. Therefore, the MEK inhibitor KZ-001 is a potent radiosensitizer for future clinical applications.
